# Personalized comfort settings in positive airway pressure machines using a causal inference approach: a retrospective observational analysis

**DOI:** 10.3389/frsle.2026.1883557

**Published:** 2026-08-21

**Authors:** Chinh Nguyen, Himani Jayawardane, Hussein Emami, Nathan Liu, Bhavana Achary, Jeff Armitstead, Alison Wimms

**Affiliations:** 1 Data Science & AI Lab, Resmed, Sydney, NSW, Australia; 2 Resmed Science Center, Sydney, NSW, Australia; 3 Resmed Science Center, Singapore, Singapore

**Keywords:** adherence, causal inference, comfort settings, continuous positive airway pressure, machine learning, obstructive sleep apnea, personalized therapy

## Abstract

**Introduction:**

To evaluate whether data–driven personalization of positive airway pressure (PAP) comfort settings improves adherence by developing and evaluating a causal machine learning (ML) model for individualized recommendations.

**Methods:**

The model was developed using AirSense 10 data and independently validated in temporally separated AirSense 10 and AirSense 11 cohorts, including 90–day and 1–year follow–up. The primary outcome was Centers for Medicare & Medicaid Services (CMS)–defined PAP adherence (device usage ≥ 4 h/night on ≥ 70% of nights during the first 90 days). Group–level average treatment effect (ATE) was estimated using backdoor adjustment methods. A causal forest model was applied to estimate the conditional average treatment effect for recommending personalized settings. A propensity score–matched analysis compared outcomes between patients whose actual settings matched model recommendations vs. those remaining on device default settings.

**Results:**

The model estimated ATE at the group level was 2.9 percentage points (p < 0.001), indicating that personalized settings were associated with improved CMS adherence. Covariate balance was achieved (standardized mean difference < 0.1), and model calibration was strong (expected calibration error 0.91%). Patients whose actual PAP settings matched the model–recommended configuration had higher device usage than matched controls who remained on default settings. Subgroup analyses confirmed consistent benefits across age groups, gender, apnea–hypopnea index, and mask type. SHapley Additive exPlanations analysis identified minimum pressure, start pressure and age as key drivers of personalization. Independent validation showed sustained usage benefits across AirSense 10 and AirSense 11 cohorts, with residual AHI remaining below the clinical reference threshold and no clinically meaningful deterioration in mask leak.

**Discussion:**

Causal ML–based personalization of PAP comfort settings was associated with improved CMS adherence/device usage. Integration of comfort setting personalization into setup workflows may enhance PAP therapy usage. As a retrospective observational analysis, these findings are associative and hypothesis–generating; the causal–inference framework, refutation testing, and large–scale independent validation provide robustness beyond that of conventional observational studies, although prospective randomized evaluation remains the definitive next step.

## 1 Introduction

Obstructive sleep apnea (OSA) is a chronic condition that is associated with negative cardiovascular, metabolic, and cognitive consequences (Patil et al., 2019). Positive airway pressure (PAP) therapy is the gold standard for treating moderate–to–severe (OSA), and mild OSA with associated symptoms and/or comorbidities (Patil et al., 2019). Although PAP is highly effective when used consistently, adherence to therapy remains a key challenge, especially during the first 30–90 days of treatment (Weaver and Sawyer, 2010; Askland et al., 2020). Failure to meet early adherence thresholds (defined by the United States [US] Centers for Medicare & Medicaid Services [CMS] as device usage of ≥ 4 h/night on ≥70% of nights during any consecutive 30–day period within the first 90 days of therapy) has been shown to predict long–term discontinuation and poor health outcomes (Malhotra et al., 2023). Specifically, adherence to PAP during the first month of therapy is strongly associated with sustained adherence at 2 years (Jeppesen et al., 2025), highlighting the critical importance of optimizing therapy at initiation.

Among the multiple factors influencing PAP therapy adherence, pressure–related discomfort is consistently noted as a major barrier (Sawyer et al., 2011; Luo et al., 2021). To address these issues, many modern PAP devices offer user–configurable settings aimed at improved comfort. These settings include expiratory pressure relief (EPR), smoothing of pressure transitions (Soft Response setting) or a gradual increase during the sleep initiation phase (Ramp mode), are intended to ease pressure transitions and reduce discomfort during sleep onset or exhalation, although their physiological effects may also influence delivered pressure dynamics and residual respiratory events in some patients. While these comfort settings are well–received by patients, there is a relative lack of evidence regarding their impact on adherence (Johnson, 2022). Comfort is, however, bidirectional. Inadequate therapeutic pressure and air hunger can themselves cause residual obstructive events and undermine adherence, so pressure–easing settings are not universally beneficial. In particular, because EPR reduces effective expiratory pressure (analogous to bilevel therapy) it may, in susceptible patients, drop expiratory pressure below the airway closing pressure and increase residual obstructive events or provoke treatment–emergent central sleep apnea. This bidirectionality further motivates a personalized rather than a one–size–fits–all approach to comfort configuration.

Individual comfort needs can vary considerably, influenced by factors including mask type, therapeutic pressure requirements, sleep patterns, and personal preference. This heterogeneity highlights the need for a personalized configuration of comfort settings (Kennedy et al., 2019; Killick and Marshall, 2021). However, in practice, comfort settings are often applied using device defaults or manually adjusted without a systematic or personalized approach. As a result, their potential benefits may not be fully realized.

The integration of comprehensive therapy and adherence data from PAP devices with advanced machine learning capabilities could facilitate the personalization of PAP therapy comfort settings. Therefore, this study applied a causal machine learning approach [combining double machine learning [DML] with a causal forest model informed by domain–specific causal diagrams that were developed using published evidence on the impact of different factors on patient–reported comfort and device usage (Sin et al., 2002; Chai et al., 2006; Chai-Coetzer et al., 2013; Cistulli et al., 2023)] to a large retrospective PAP therapy dataset. We implemented and evaluated this causal machine learning model to recommend personalized comfort settings for PAP therapy (ComfortMatch, SmartComfort, Resmed). We also investigated whether these recommendations could improve CMS–defined adherence and therapy usage.

## 2 Materials and methods

### 2.1 Study design and cohort selection

This retrospective observational analysis reviewed home telemonitoring data from de–identified patients with OSA from the US who received PAP therapy with an AirSense 10 device (Resmed) between May 25, 2016, and July 26, 2021. Data were collected using Resmed's cloud–based platform (Air View), which continuously captures device settings and usage metrics, and the accompanying patient engagement app (my Air), which records demographic and mask–related data.

Analysis and model development utilized data from new PAP users ( ≤ 3 months of prior use) who exclusively used the AutoSet algorithm, did not modify therapy or comfort settings within the first 90 days (patients who kept their initial settings are retained irrespective of CMS adherence), and used one of nine predefined comfort setting combinations. Patients were excluded if they used other devices or modes, had an average mask leak >25 L/min, or were missing data for key demographic, device setting, or outcome variables.

Independent validation was performed using retrospective datasets that were not used during model development. These datasets included patients using Air Sense 10 and Air Sense 11 devices with device set–up dates from October 2022 to May 2025, and included approximately 1.27 million patients with 90–day follow–up and approximately 1.28 million patients with 1–year follow–up. The validation datasets were used to evaluate temporal generalizability, generalizability to newer–generation Resmed hardware, and longer–term usage and efficacy endpoints. These cohorts were temporally and/or device–generation separated from the original development cohort.

### 2.2 Comfort setting variables (interventions)

The primary interventions reviewed in this analysis were the adjustable comfort settings available on Resmed AirSense 10 PAP devices, specifically Soft Response Mode, Ramp Mode, Expiratory Pressure Relief (EPR) Mode, and EPR Level. These configurable options are designed to enhance patient comfort without altering therapeutic pressure targets and were modeled as categorical treatment combinations for causal analysis. Specific details of these modes are as follows:

- Soft Response Mode: adjusts the behavior of the AutoSet algorithm. When enabled (Soft), pressure increases more gradually in response to respiratory events, potentially improving sleep continuity in sensitive individuals.
- Ramp Mode (Auto, On, or Off): starts therapy at a lower pressure to improve comfort during sleep onset. In Auto mode, the device detects sleep onset and increases pressure automatically (Ramp Time is not applicable). In On mode, pressure rises over a fixed Ramp Time set by the user or clinician. Off mode starts therapy at the full prescribed pressure.
- EPR Mode (Off, Ramp Only, or Full Time): this determines whether EPR is applied and for how long during the therapy session.
- EPR Level (1, 2, or 3 cmH_2_O): specifies the magnitude of pressure relief during exhalation.

The analysis was limited to nine predefined comfort setting combinations selected during model development (Table 1). These configurations were chosen by clinical experts and data scientists to represent distinct, interpretable combinations of Soft Response Mode, Ramp Mode, EPR Mode and EPR Level, and to ensure sufficient sample sizes for reliable modeling within each group. Although >100 possible combinations were present in the raw dataset, many were rare or clinically redundant. The nine selected configurations accounted for approximately 70% of all patients in the dataset and captured the clinically meaningful variation in comfort features relevant to outcome measures.

**Table 1:** Details of the nine predefined comfort settings and their distribution in the training dataset (Air Sense 10 device).

Settings combinations					Proportion, %
Soft response	RAMP mode	RAMP time	EPR mode	EPR level	
Off	Auto	–	Full–time	3	24.78
Off	Off	N/A	Full–time	3	13.93
Off	Auto	–	Full–time	2	11.20
Off	Off	N/A	Full–time	2	6.83
Off	Off	N/A	Off	N/A	4.84
Off	Auto	–	Full–time	1^*^	3.76
Off	Off	N/A	Full–time	1	1.94
On	Off	N/A	Full–time	3	1.90
On	Off	N/A	Full–time	2	0.87
Coverage					**70.05**

The combination OFF_AUTO_NA_FULLTIME_THREE represents a standard pressure response, auto ramp enabled (no fixed ramp time), and full–time EPR at level 3. ^*^Device default settings. EPR, expiratory pressure relief; N/A, not applicable.

### 2.3 Outcome measures

The primary outcome was CMS adherence (PAP device usage of ≥4 h/night on ≥70% of nights during any consecutive 30–day period within the first 90 days of therapy). Secondary outcomes included the average usage duration (in min/night) and the average number of days with any recorded device usage during the first 90 days of therapy. The independent validation additionally evaluated residual apnea–hypopnea index (rAHI) as an objective efficacy measure (with a pre–specified clinical reference of < 5 events/h) and average mask leak at both 90 days and 1 year to assess durability and potential efficacy

### 2.4 Feature sets and confounders

The analysis and model incorporated a set of mandatory input features representing baseline patient characteristics and therapy configurations at setup:

- Age group: < 45, 45–60, or >60 years.
- Gender: self–reported as female, male, or prefer not to say.
- Type of sleep test: home sleep test or in–laboratory polysomnography (PSG) used in the diagnostic pathway.
- Baseline AHI group: self–reported apnea–hypopnea index (AHI) of < 5 events/h (minimum), 5 to < 15 events/h (mild), 15 to < 30 events/h (moderate), ≥30 events/h (severe), or unknown.
- Mask type: interface used during PAP therapy (nasal, nasal pillows, or full–face mask).
- Pre–therapy sleepiness: self–reported daytime sleepiness before PAP initiation (rated on a scale from ‘None' ‘slight', ‘moderate', ‘very', ‘Extremely', or Unknown.
- Minimum pressure: the minimum pressure device setting at therapy initiation (from 4.0 to 20.0cmH_2_O, in increments of 0.2cmH_2_O).
- Start pressure: starting pressure (used with Ramp; ranging from 4.0 to 20.0cmH_2_O, in increments of 0.2cmH_2_O).

The non–mandatory features (baseline AHI group and pre–therapy sleepiness) were not statistically imputed but were encoded with an explicit “Unknown” ordinal category. Baseline AHI was self–reported, and pre–therapy sleepiness was missing (recorded as “Unknown”) in approximately 60% of patients. This missingness arose predominantly from non–completion of the optional myAir app onboarding survey rather than from clinical status, supporting a missing–completely–at–random or missing–at–random mechanism, and the explicit “Unknown” category retained these patients within the adjustment set without imposing imputation assumptions. Although an informative (missing–not–at–random) mechanism cannot be formally excluded, its practical impact appeared limited: in the Monte Carlo input–misclassification analysis, perturbing these self–reported inputs produced low top−1 recommendation flip rates (4.7% for baseline AHI and 1.4% for pre–therapy sleepiness), and a sensitivity analysis excluding patients with baseline AHI < 5 events/h preserved the direction, significance, and approximate magnitude of the estimated treatment effects.

### 2.5 Causal inference approach

#### 2.5.1 Causal diagram

**Figure 1:**
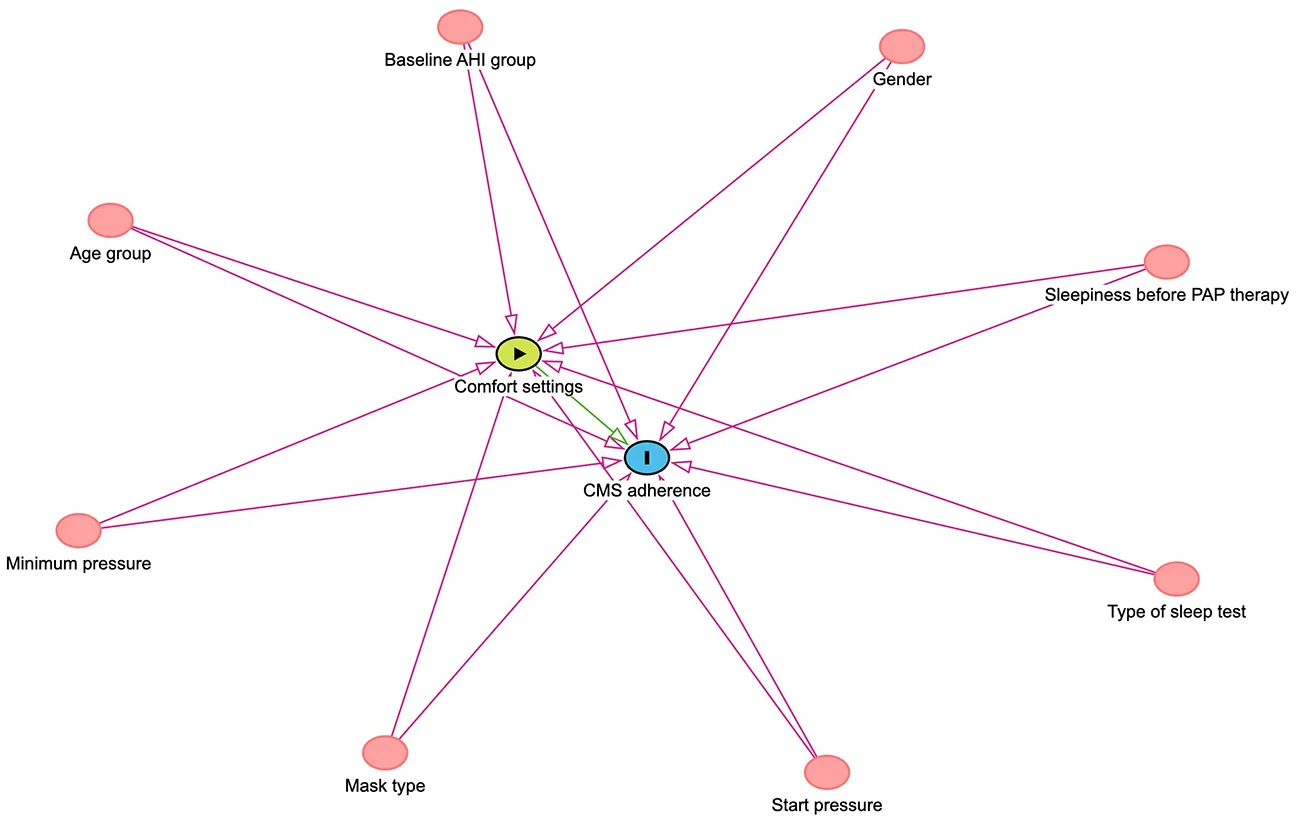
Causal diagram showing the hypothesized relationships between the treatment (comfort settings), outcome (CMS adherence) and patient input features. AHI, apnea–hypopnea index; CMS, Centers for Medicare & Medicaid Services; PAP, positive airway pressure.

A causal diagram, or directed acyclic graph (DAG), was constructed to visually represent the hypothesized relationships between comfort settings (treatment), CMS adherence (outcome), and patient input features (covariates) (Figure 1). The DAG explicitly defines potential confounding variables such as age, gender, OSA severity, mask type, and pressure settings. Note that minimum pressure and start pressure are positioned as pre–treatment confounders (prescribed before comfort settings are determined and not modified as a consequence of comfort–setting assignment) rather than as mediators, so adjusting for them does not induce collider bias.

This structured representation informed variable selection and guided adjustment strategies to ensure that causal estimates were not biased by backdoor paths (non–causal associations arising from confounding). By identifying an appropriate backdoor adjustment set, we estimated the average treatment effect (ATE) at the group level using standard covariate adjustment methods (e.g., regression–based and propensity score–based approaches). In parallel, we developed a causal machine learning framework based on DML and causal forests to estimate the conditional average treatment effect (CATE) at the individual patient level, enabling personalized comfort setting recommendations tailored to each patient's baseline profile. This dual approach allowed assessment of both the population–level impact and the individualized benefit of comfort personalization.

#### 2.5.2 Estimation of ATE (group level)

To estimate the ATE of comfort settings on CMS adherence at the population level, we applied the backdoor adjustment criterion (11, 12). The causal diagram facilitated the identification of a valid backdoor adjustment set, a group of baseline covariates sufficient to block all non–causal pathways between the treatment and the outcome.

To ensure robustness and validate the causal effect of comfort settings on adherence, four standard backdoor adjustment methods implemented in the DoWhy framework were applied: linear regression; propensity score matching; propensity score stratification; and propensity score weighting. The linear regression adjustment method estimates treatment effects by modeling the outcome as a linear function of the treatment variable and covariates, and can be represented as follows:


$$\begin{array}{l} \text{CMS adherance }\sim \text{ Comfort settings}+\text{Age group}\\ +\text{ Start pressure}+\text{Baseline AHI group}\\ +\text{ Type of sleep test}+\text{Gender}+\text{Minimum pressure}\\ +\text{ Sleepiness before PAP therapy}+\text{Mask type} \end{array}$$

The effect of *Comfort settings* on *CMS adherance* was estimated while adjusting for baseline covariates identified through the backdoor criterion, isolating the treatment effect by controlling for potential confounding. In addition, distance matching (which compares outcomes between treated and control units with similar covariates), propensity score stratification (which divides the sample into subgroups based on the estimated probability of receiving treatment and compares outcomes within these strata), and propensity score weighting (which reweights individuals using the inverse probability of treatment to balance covariates across treatment groups) were applied. Together, these methods provide complementary strategies for estimating the ATE and collectively strengthen the credibility of the group–level conclusions.

#### 2.5.3 Estimation of CATE (patient level)

**Figure 2:**
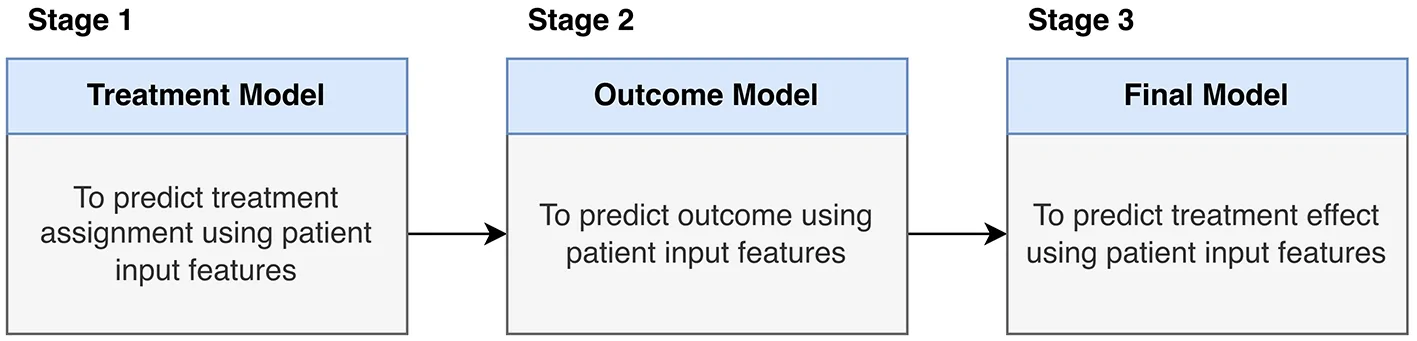
The three model training stages used during development of the personalized recommendation engine.

While group–level ATE estimation provides population–wide insights, CATE estimation at the patient level enables personalized comfort setting recommendations tailored to the characteristics of each individual. To estimate the CATE, we applied a DML framework using a causal forest model as the final estimator (Figure 2). This approach estimates heterogeneous treatment effects while adjusting for observed confounders, supporting individualized treatment decisions. The estimation involved the following steps:

1. Nuisance Model Training: two ML models were trained as follows.
  - A propensity model to estimate the probability of assignment to each comfort setting combination based on patient covariates.
  - An outcome model to predict the CMS adherence from the same patient covariates, independent of treatment assignment.
2. Residualization and causal forest estimation: residuals from the nuisance models were computed and passed into the causal forest model, which learns how treatment effects vary between subpopulations. This approach uses tree–based splits to detect treatment heterogeneity, estimating an individualized CATE for each patient across all feasible comfort setting combinations.
3. Personalized recommendation: the model generated predicted probability of adherence under each comfort setting combination for each patient. The setting with the best predicted outcome was selected as the recommended comfort configuration. This enabled a data–driven, patient–specific recommendation for optimizing CMS adherence at the individual level.

The causal forest model served as the final model supporting the personalized recommendation engine (PRE) evaluated in this analysis.

To aid interpretability, patient–level recommendation outputs were subsequently examined using SHapley Additive exPlanations (SHAP)–based explanations, which quantify the relative contribution of individual patient features to each predicted recommendation.

### 2.6 Evaluation methods

#### 2.6.1 Refutation tests for ATE estimation

To assess the robustness of the causal effect estimates, a series of refutation tests was conducted using the DoWhy framework. These tests introduce controlled perturbations to the data or model assumptions to evaluate the sensitivity of the estimated ATE to potential violations of key assumptions (e.g., unmeasured confounding, model misspecification, or random noise). Specifically, the following refutation tests were performed: (1) random common cause (introduces a randomly generated variable as a confounder to test whether the estimated effect changes significantly, indicating sensitivity to hidden variables); (2) placebo treatment (replaces the actual treatment variable with a randomly permuted version to determine whether the observed effect persists when treatment is unrelated to the outcome); and (3) data subset refuter (repeats the estimation on a random subsample of the data to examine the stability of results across different population subsets).

#### 2.6.2 Model evaluation metrics

These were standardized mean difference (SMD) for the treatment model, expected calibration error (ECE) for the outcome model, and best linear predictor (BLP) for the final model (Supplementary Figure 5, Supplementary Figure 6, Supplementary Table 1).

#### 2.6.3 PRE evaluation on test data

PRE was evaluated on a held–out test dataset by comparing outcomes (i.e., CMS adherence and therapy usage) between patient groups defined by their alignment with the model's comfort setting recommendations.

In the first experiment, the treatment group included patients whose actual comfort settings matched the model's recommended configuration and the control group included patients who remained on the default device settings despite the model recommending an alternative. This evaluation approximated the effect of following the model's recommendations compared with maintaining default settings.

In the second experiment, the treatment group of patients whose actual comfort settings matched the model's recommended configuration was compared with a different control group that included patients whose actual comfort settings differed from both the device defaults and the model's recommendations. This additional comparison isolated the impact of the PRE from clinician– or patient–driven adjustments.

To ensure comparability, all groups were matched using propensity score matching based on baseline patient covariates.

The same group definitions were then applied to the independent validation datasets stratified by device generation and follow–up duration. The corresponding device–specific default comfort configuration was used as the reference: OFF_AUTO_NA_FULLTIME_ONE for AirSense 10 and OFF_AUTO_NA_RAMP_ONLY_ONE for AirSense 11.

#### 2.6.4 Sensitivity analyses

Several sensitivity analyses were conducted to evaluate robustness of the findings. First, to assess potential misclassification from self–reported baseline AHI, treatment–effect estimates were recalculated after excluding patients with baseline AHI < 5 events/h. Second, To assess robustness to real–world input uncertainty, a Monte Carlo input–misclassification sensitivity analysis was performed on the held–out test set. Feature–specific perturbation models were defined based on expected input reliability. Baseline AHI group and pre–PAP sleepiness were assigned a 20% ordinal misclassification rate, reflecting their greater uncertainty as self–reported variables. Mask type was assigned a 4% nominal misclassification rate; sleep test type and age group were assigned 2% misclassification rates; and gender was assigned a 0.3% misclassification rate. Minimum pressure and start pressure were perturbed using Gaussian jitter with SD = 0.4 cmH_2_O plus a 1% gross–entry error rate. One–at–a–time perturbation analyses were first used to identify influential inputs, followed by a joint Monte Carlo analysis in which all eight inputs were perturbed simultaneously across 500 replications. Recommendation stability was summarized using the top−1 recommendation flip rate. Third, to evaluate potential selection bias from restricting the analysis to nine predefined comfort–setting combinations, selected and not–selected patients were compared across measured baseline characteristics, and treatment effects were re–estimated in a restricted cohort using only the four most prevalent comfort–setting combinations.

### 2.7 Statistical analysis

All statistical analyses were conducted using Python (v3.10) with packages including scikit–learn, econml, DoWhy, and statsmodels. For all comparisons, a two–sided *p*–value of < 0.05 was considered statistically significant. Model calibration was assessed using SMD, ECE and BLP. Subgroup analyses and HAP–based feature attributions were used for exploratory validation and interpretability.

## 3 Results

**Figure 3:**
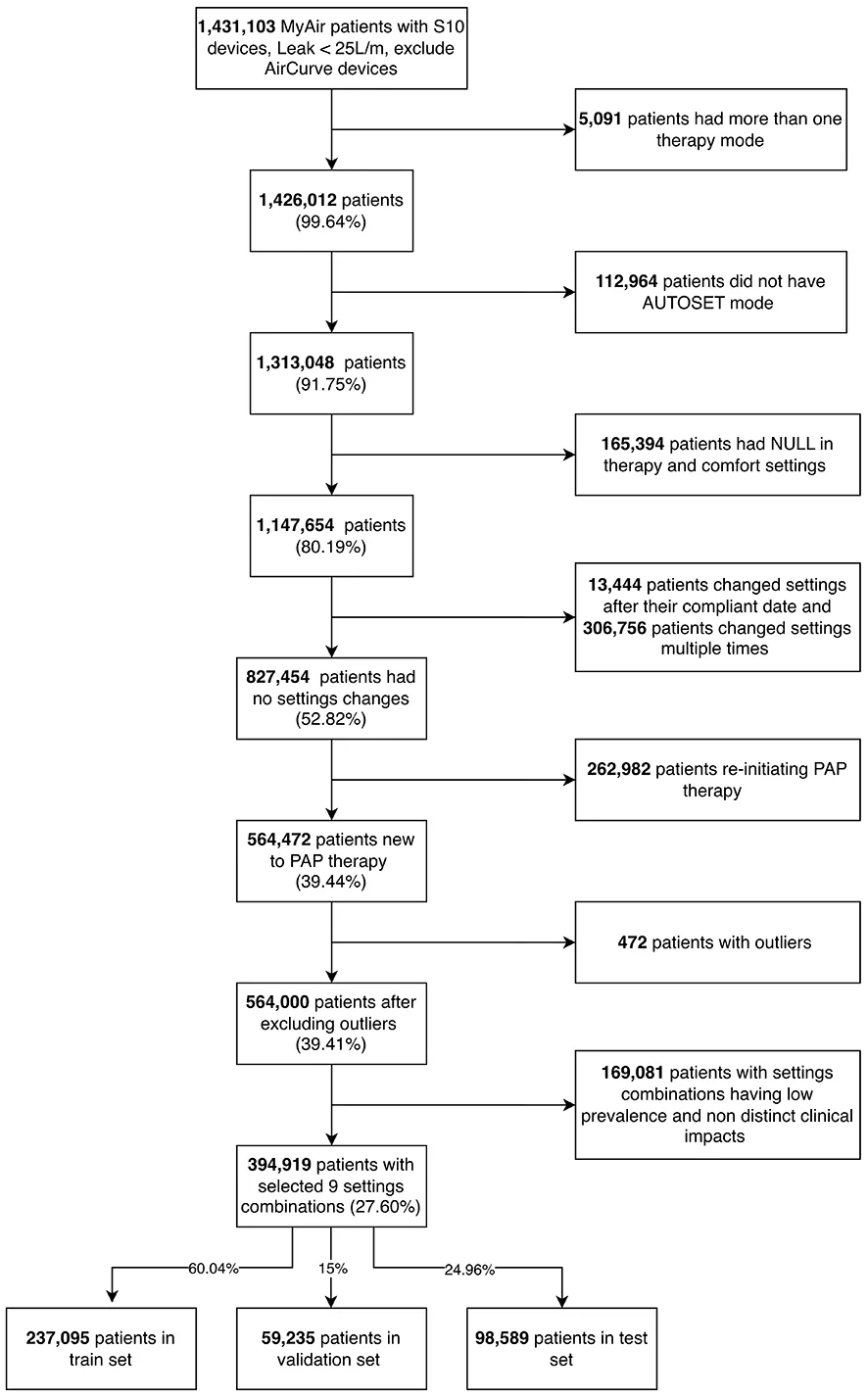
Study flow chart. PAP, positive airway pressure therapy.

Of 1,431,103 eligible patients receiving PAP therapy with an AirSense 10 device, 394,919 met all inclusion criteria and had PAP therapy settings that matched the nine predefined setting combinations (Figure 3). These participants were divided into training (*n* = 237, 095), validation (*n* = 59, 235), and test (*n* = 98, 589) sets. Patient characteristics were comparable between these three sets (Table 2).

**Table 2:** Patient characteristics.

Characteristic	Category	Overall	Train set	Validation set	Test set	*p*–value
Age group, *n* (%)	< 45 years	122,745 (31.1)	73,704 (31.1)	18,454 (31.2)	30,587 (31)	0.594
	45–60 years	104,935 (26.6)	62,818 (26.5)	15,843 (26.7)	26,274 (26.7)	
	>60 years	167,239 (42.3)	100,573 (42.4)	24,938 (42.1)	41,728 (42.3)	
Gender, *n* (%)	Female	132,355 (33.5)	79,646 (33.6)	19,847 (33.5)	32,862 (33.3)	0.616
	Male	234,126 (59.3)	140,442 (59.2)	35,126 (59.3)	58,558 (59.4)	
	Prefer not to say	28,438 (7.2)	17,007 (7.2)	4,262 (7.2)	7,169 (7.3)	
Baseline AHI group, *n* (%)	< 5 events/h	73,887 (18.7)	44,301 (18.7)	11,192 (18.9)	18,394 (1)	0.367
	5 to < 15 events/h	105,156 (26.6)	62,984 (26.6)	15,709 (26.5)	26,463 (26.8)	
	15 to < 30 events/h	77,532 (19.6)	46,616 (19.7)	11,630 (19.6)	19,286 (19.6)	
	≥30 events/h	87,533 (22.2)	52,795 (22.3)	12,986 (21.9)	21,752 (22.1)	
	Unknown	50,811 (12.9)	30,399 (12.8)	7,718 (13.0)	12,694 (12.9)	
Mask type, *n* (%)	Full face	159,005 (40.3)	95,418 (40.2)	23,911 (40.4)	39,676 (40)	0.783
	Nasal	106,896 (27.1)	64,100 (27.0)	16,107 (27.2)	26,689 (27.1)	
	Pillows	129,018 (32.7)	77,577 (32.7)	19,217 (32.4)	32,224 (32.7)	
Type of sleep test, *n* (%)	Home	266,495 (67.5)	159,948 (67.5)	39,934 (67.4)	66,613 (67.6)	0.786
	Sleep laboratory	128,424 (32.5)	77,147 (32.5)	19,301 (32.6)	31,976 (32.4)	
Level of self–reported daytime sleepiness before starting PAP therapy, *n* (%)	Extreme	13,823 (3.5)	8,329 (3.5)	2,086 (3.5)	3,408	0.489
	Very	35,274 (8.9)	21,191 (8.9)	5,192 (8.8)	8,891 (9.0)	
	Moderate	66,004 (16.7)	39,466 (16.6)	10,003 (16.9)	16,535 (16.8)	
	Slight	32,161 (8.1)	19,248 (8.1)	4,932 (8.3)	7,981 (8.1)	
	None	10,627 (2.7)	6,424 (2.7)	1,581 (2.7)	2,622 (2.7)	
	Unknown	237,030 (60.0)	142,437 (60.1)	35,441 (59.8)	59,152 (60.0)	
Mean minimum pressure, cmH_2_O		5.4 ± 1.5	5.4 ± 1.5	5.3 ± 1.5	5.3 ± 1.5	0.475
Mean start pressure, cmH_2_O		4.2 ± 0.6	4.2 ± 0.6	4.2 ± 0.6	4.2 ± 0.6	0.205
CMS adherence, *n* (%)	No	79,689 (20.2)	47,851 (20.2)	11,912 (20.1)	19,926 (20.2)	0.886
	Yes	315,230 (79.8)	189,244 (79.8)	47,323 (79.9)	78,663 (79.8)	

Values are mean ± standard deviation, or number of patients (%). AHI, apnea–hypopnea index.

### 3.1 Effect of comfort settings on patient outcomes (group level)

The ATE was consistently positive across all four backdoor adjustment methods, indicating that, on average, the use of alternative comfort settings was associated with a higher likelihood of treatment adherence compared with the default setting. Estimates ranged from 0.015 (distance matching) to 0.030 (propensity score weighting), with linear regression and propensity score stratification yielding intermediate values of 0.029 and 0.027, respectively. Despite minor differences in magnitude due to methodological variations, the consistent direction and effect size across methods suggest a robust positive causal effect whereby the use of alternative comfort settings was associated with an approximately 3% increase in the likelihood of achieving CMS adherence. The causal machine learning framework treated the nine comfort–setting combinations as distinct treatment arms, enabling estimation of each non–default setting's effect relative to the AirSense 10 default setting. Treatment–arm–specific summaries derived from the individual–level CATE estimates, including mean effects and 95% confidence intervals, are shown for comparison (Supplementary Table 2).

#### 3.1.1 Refute tests

Adding a random common cause had no impact on the ATE (0.0290 before vs. 0.0290 after; *p* = 1.0), suggesting that unmeasured confounding was unlikely to explain the observed effect. When the actual treatment assignment was replaced with a randomly permuted version, the estimated effect dropped to nearly zero (*p* = 0.98), reinforcing the conclusion that the original result was not due to randomness. Similarly, repeating the analysis on a random subset of the data produced a nearly identical effect (0.0293; *p* = 0.88), indicating that the findings were not sensitive to sample variation.

### 3.2 Effect of comfort settings on patient outcomes (patient level)

**Figure 4:**
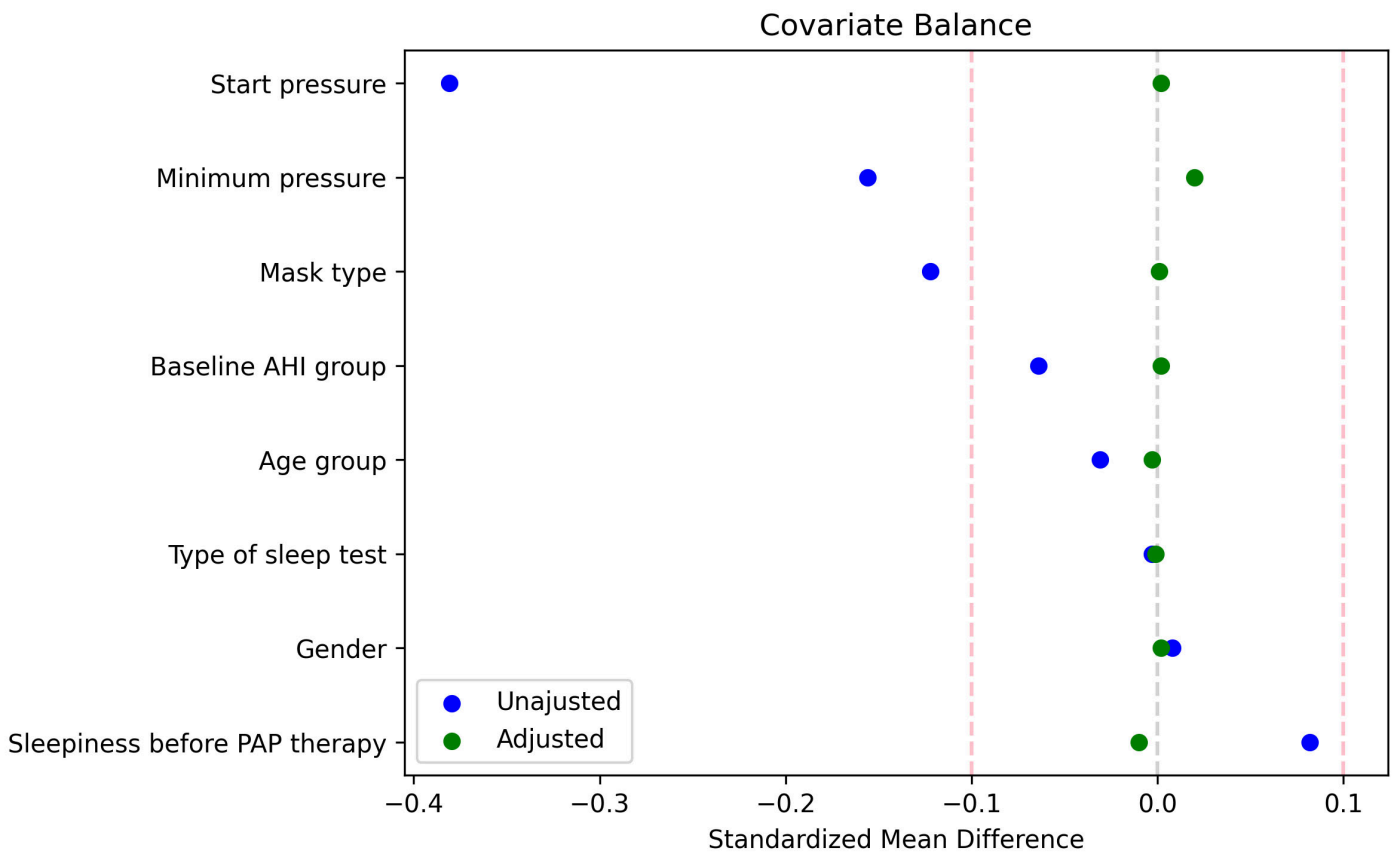
Covariate balance measured by standardized mean difference before (unadjusted) and after (adjusted) propensity score matching for primary analysis.

The first experiment included 5,875 patients whose actual comfort settings aligned with the model's recommendations (treatment group) and 5,339 patients who remained on the default settings despite receiving an alternative recommendation (control group). ATE showed that the treatment group had an approximate 4% increase in adherence, a –12 min/night increase in device usage, and an additional 2 days of PAP usage in the first 90 days of therapy compared with the control group (all *p* < 0.001) (Table 3, Figure 4). The corresponding *E*–values suggest that an unmeasured confounder would need to be *moderately associated* with both treatment assignment and outcome to fully explain the observed effects.

**Table 3:** Average treatment effect against Air Sense 10 default settings.

Outcome	*ATE*	*95% CI*	*p*–value	*E*–value
CMS adherence	0.0454	0.0247, 0.0661	< 0.0001	1.2190
PAP usage, min/night	12.19	6.87, 17.51	< 0.0001	1.3886
PAP usage, days used in the first 90 days	2.76	1.48, 4.05	< 0.0001	1.3008

ATE, average treatment effect; CI, confidence interval; CMS, Centers for Medicare & Medicaid Services.

The second experiment included the same treatment group (5,875) and a control group of 87,375 patients whose actual comfort settings differed from both the device defaults and the model's recommendations. ATE showed that the treatment group had an approximate 3.7% increase in adherence, used their PAP device for –7 min longer per night, and had an additional 2 days of PAP usage within the first 90 days of therapy compared with the control group (all *p* < 0.001) (Table 4 and Supplementary Figure 1). The corresponding E–values suggest that an unmeasured confounder would need to be moderately to strongly associated with both treatment assignment and outcome to fully account for the observed effects.

**Table 4:** Average treatment effect against clinician– or patient–adjusted settings.

Outcome	ATE	95% CI	*p*–value	*E*–value
CMS adherence	0.0365	0.0229, 0.0500	< 0.0001	1.2544
PAP usage, min/night	7.61	4.10, 11.12	< 0.0001	1.4502
PAP usage, days used in the first 90 days	2.02	1.17, 2.87	< 0.0001	1.3300

ATE, average treatment effect; CI, confidence interval; CMS, Centers for Medicare & Medicaid Services.

In the independent validation analysis comparing recommendation–matched comfort settings with device–specific defaults, usage benefits were observed across both Air Sense 10 and Air Sense 11 cohorts and were generally sustained at 1 year. In the Air Sense 10 cohort, the analysis included 7,036 treatment and 4,767 control patients at 90 days, and 4,073 treatment and 2,137 control patients at 1 year. Patients showed + 8.4 min/night at 90 days (*p* = 0.0003), + 8.0 min/night at 1 year (*p* = 0.0036), and + 12.3 additional usage–days over 1 year (*p* < 0.001) (Table 5). Effects were larger and more consistent in the AirSense 11 cohort, which included 47,981 treatment and 11,187 control patients at 90 days, and 21,656 treatment and 3,954 control patients at 1 year. Patients showed + 20.4 min/night at 90 days (*p* < 0.001), + 15.2 min/night at 1 year (*p* < 0.001), and + 21.2 additional usage–days at 1 year (*p* < 0.001) (Table 6). In the independent validation analysis comparing recommendation–matched comfort settings with clinician– or patient–adjusted non–default settings, usage benefits were again observed across both Air Sense 10 and Air Sense 11 cohorts and were sustained at 1 year. In the Air Sense 10 cohort, the analysis included 109,809 treatment and 4,767 control patients at 90 days, and 64,287 treatment and 2,137 control patients at 1 year. Patients showed + 8.1 min/night at 90 days (*p* < 0.001), + 7.1 min/night at 1 year (*p* = 0.0010), + 1.8 additional usage–days at 90 days (*p* = 0.0001), and + 11.7 additional usage–days over 1 year (*p* < 0.001) (Table 7). Effects were also consistent in the Air Sense 11 cohort, which included 382,061 treatment and 11,187 control patients at 90 days, and 182,329 treatment and 3,954 control patients at 1 year. Patients showed + 13.1 min/night at 90 days (*p* < 0.001), +13.6 min/night at 1 year (*p* < 0.001), + 3.2 additional usage–days at 90 days (*p* < 0.001), and + 19.1 additional usage–days over 1 year (*p* < 0.001) (Table 8).

**Table 5:** Average treatment effect against AirSense 10 default settings (independent validation).

Outcome	Duration	ATE	95% CI	*p*–value	*E*–value
PAP usage, min/night	90 days	8.37	3.81, 12.93	0.0003	1.3106
PAP usage, min/night	1 year	8.04	2.62, 13.47	0.0036	1.8271
PAP usage, days used in the first 90 days	90 days	1.05	−0.04, 2.15	0.0599	1.5022
PAP usage, days used in the first 90 days	1 year	12.34	7.45, 17.23	< 0.0001	1.7465
rAHI	90 days	0.29	0.14, 0.45	0.0002	1.5070
rAHI	1 year	0.09	−0.02, 0.21	0.1174	1.3244
Mask leak	90 days	0.002	−0.003, 0.007	0.3918	1.4201
Mask leak	1 year	0.003	−0.004, 0.011	0.3703	1.2050

ATE, average treatment effect; CI, confidence interval; rAHI, residual apnea–hypopnea index.

**Table 6:** Average treatment effect against Air Sense 11 default settings (independent validation).

Outcome	Duration	ATE	95% CI	*p*–value	*E*–value
PAP usage, min/night	90 days	20.40	17.32, 23.49	< 0.0001	1.5326
PAP usage, min/night	1 year	15.18	11.41, 18.95	< 0.0001	1.8173
PAP usage, days used in the first 90 days	90 days	5.80	5.03, 6.57	< 0.0001	1.5696
PAP usage, days used in the first 90 days	1 year	21.21	17.88, 24.56	< 0.0001	2.0778
rAHI	90 days	0.35	0.27, 0.44	< 0.0001	1.5175
rAHI	1 year	0.14	0.06, 0.22	0.0004	1.4153
Mask leak	90 days	−0.011	−0.014, −0.008	< 0.0001	1.3365
Mask leak	1 year	−0.009	−0.014, −0.004	0.0005	1.3112

ATE, average treatment effect; CI, confidence interval; rAHI, residual apnea–hypopnea index.

**Table 7:** Average treatment effect against clinician– or patient–adjusted settings Air Sense 10 (independent validation).

Outcome	Duration	ATE	95% CI	*p*–value	*E*–value
PAP usage, min/night	90 days	8.09	4.40, 11.78	< 0.0001	1.3903
PAP usage, min/night	1 year	7.0930	2.88, 11.31	0.0010	1.3533
PAP usage, days used in the first 90 days	90 days	1.8002	0.93, 2.67	0.0001	1.5335
PAP usage, days used in the first 90 days	1 year	11.68	7.99, 15.36	< 0.0001	1.7183
rAHI	90 days	−0.07	−0.21, 0.07	0.3298	1.0823
*rAHI*	1 year	−0.15	−0.24,−0.07	0.0005	1.4688
Mask leak	90 days	0.028	0.024, 0.032	< 0.0001	1.8857
Mask leak	1 year	0.029	0.023, 0.035	< 0.0001	1.9241

ATE, average treatment effect; CI, confidence interval; rAHI, residual apnea–hypopnea index.

**Table 8:** Average treatment effect against clinician– or patient–adjusted settings AirSense 11 (independent validation).

Outcome	Duration	ATE	95% CI	*p*–value	*E*–value
PAP usage, min/night	90 days	13.06	10.37, 15.76	< 0.0001	1.3555
PAP usage, min/night	1 year	13.63	10.19, 17.07	< 0.0001	1.3864
PAP usage, days used in the first 90 days	90 days	3.17	2.51, 3.83	< 0.0001	1.4217
PAP usage, days used in the first 90 days	1 year	19.08	16.13, 22.04	< 0.0001	1.6305
rAHI	90 days	−0.05	−0.12, 0.03	0.2155	1.1501
rAHI	1 year	−0.13	−0.21, −0.05	0.0018	1.3794
Mask leak	90 days	0.024	0.021, 0.027	< 0.0001	1.7981
Mask leak	1 year	0.029	0.024, 0.033	< 0.0001	1.8936

ATE, average treatment effect; CI, confidence interval; rAHI, residual apnea–hypopnea index.

The model is designed and validated to improve PAP therapy adherence as measured by CMS compliance criteria. The model is not validated to optimize residual AHI, sleep architecture, subjective sleepiness, or broader health outcomes. Although improved adherence is associated with better clinical outcomes at the population level, increased device use alone does not necessarily confirm therapeutic benefit for an individual patient, which remains dependent on adequate control of residual disease and patient–specific clinical response. To evaluate potential trade–offs in therapeutic efficacy or interface performance, we examined residual AHI and mask leak in the independent validation analyses. Compared with device–specific defaults, recommendation–matched settings were associated with small increases in residual AHI in some comparisons; however, mean residual AHI remained well below the clinical reference threshold of 5 events/h at both 90 days and 1 year. Compared with clinician– or patient–adjusted non–default settings, residual AHI was similar at 90 days and modestly lower at 1 year in both Air Sense 10 and Air Sense 11 cohorts. Mask leak was unchanged in the Air Sense 10 default comparison and marginally lower in the Air Sense 11 default comparison, although it was slightly higher when compared with clinician– or patient–adjusted non–default settings. Overall, these findings indicate that adherence gains were achieved while maintaining stable therapeutic efficacy and interface performance at the population level, and underscore that clinical review of residual events, leak, symptoms, patient tolerance, and other individual factors remains important when applying comfort–setting recommendations (Tables 5–8).

### 3.3 Sensitivity analysis

Sensitivity analyses supported the robustness of the primary findings. Excluding patients with baseline AHI < 5 preserved the direction, statistical significance, and approximate magnitude of the estimated treatment effects in both experiments (Supplementary Table 3). Compared with AirSense 10 default settings, recommendation–matched settings remained associated with higher CMS adherence, longer nightly usage, and more usage days. Similar findings were observed when recommendation–matched settings were compared with clinician– or patient–adjusted non–default settings.

In the input–misclassification sensitivity analysis, start pressure and minimum pressure were the most influential inputs, producing top−1 recommendation flip rates of 13.1% and 10.2%, respectively, in one–at–a–time perturbation analyses. Self–reported baseline AHI group and pre–therapy sleepiness had smaller effects, with flip rates of 4.7% and 1.4%, respectively, while all remaining inputs produced < 1% recommendation instability. In the joint Monte Carlo analysis, simultaneous perturbation of all eight inputs produced a median top−1 recommendation flip rate of 24.2% across 500 replications, corresponding to 75.8% recommendation stability. Most changes occurred between closely related comfort–setting configurations, suggesting that the flip rate primarily reflected top−1 label sensitivity rather than large shifts between clinically dissimilar recommendations.

Sensitivity analyses were conducted to assess the potential impact of cohort selection and underrepresentation of less common comfort–setting combinations (Supplementary Table 4). Selected and not–selected patients were broadly similar across measured baseline characteristics, with differences in covariate proportions generally small and mean pressure settings differing by < 0.2 cmH_2_O. In a restricted analysis using only the four most prevalent comfort–setting combinations, compliance and usage–day effects remained directionally consistent with the primary analysis, although estimates were less precise and did not reach statistical significance, likely reflecting the substantially smaller treated sample size (Table 5). These findings suggest that the primary results were not driven by large observable differences between selected and excluded patients, while confirming greater uncertainty for less prevalent comfort–setting combinations.

### 3.4 Feature importance by SHAP values

#### 3.4.1 Group level

The top three features (minimum pressure, start pressure, and age) consistently showed the greatest impact (Supplementary Figure 2).

#### 3.4.2 Patient level

Based on data for two sample patients, two different approaches to therapy were recommended (Supplementary Figure 3). For a patient with severe baseline OSA (AHI ≥30 events/h), a high level of baseline sleepiness, and a high minimum pressure (9cm H_2_O), the model recommended enabling Soft Response to allow more gradual pressure increases during respiratory events, helping maintain sleep continuity despite the higher pressure. With Ramp Mode turned off, therapy begins immediately at the prescribed pressure, supporting rapid and sustained intervention for severe OSA. EPR is set to *Full Time* with a moderate relief level of 2, enhancing comfort during exhalation and reducing potential discomfort from elevated inspiratory pressures.

For a patient with mild OSA at baseline (AHI 5 to < 15 events/h), younger age (< 45 years), and a low minimum pressure (4 cmH_2_O), the model recommended simplified settings with limited comfort intervention. Soft Response is turned off, as additional pressure smoothing is not required at this low therapeutic pressure. EPR is enabled full–time at 1 to provide mild expiratory relief providing comfort during exhalation and encouraging sustained device use. Ramp Mode is turned off, allowing therapy to begin immediately at the prescribed pressure, which is appropriate given the low pressure and the patient's younger age. Additionally, the use of a home sleep test–based diagnostic pathway contributed to a more conservative and streamlined configuration.

To make the model outputs interpretable at a glance, the dominant recommended comfort configuration was summarized across key feature combinations (Supplementary Table 6). The model stratifies primarily by prescribed start pressure: patients with the lowest pressures require no EPR, whereas those at higher pressures are assigned EPR level 2–3 to improve exhalation tolerability; Soft Response is additionally favored for younger patients and those with unknown severity (a conservative choice when tolerance is less predictable), and lab–diagnosed patients tend to receive EPR without Soft Response, consistent with more clinician–guided management.

### 3.5 Subgroup analysis

The substantial overlap observed between the distributions of CATE estimates for different subgroups (Supplementary Figure 4) suggests that the estimated treatment effects are not markedly distinct, indicating broadly similar causal impacts across these key patient subgroups/characteristics.

## 4 Discussion

This analysis showed that applying a causal machine learning framework to personalize PAP comfort settings has the potential to improve PAP adherence and device usage. At both the population and individual levels, model–recommended comfort setting configurations were associated with higher device usage than device default settings, suggesting that precision adjustments based on individual characteristics could lead to better adherence to PAP therapy. Importantly, this approach aims to determine which comfort settings are most likely to improve an individual's likelihood of meeting CMS adherence criteria, which extends beyond traditional prediction tasks. Unlike standard predictive models, which estimate the probability of an outcome based on its associations with patient characteristics, the DML with a causal forest model approach estimates counterfactual outcomes (i.e., what would happen to adherence in a given individual if a different comfort setting had been applied). This enabled us to estimate CATEs and generate personalized recommendations based on baseline characteristics for each patient. In addition to individual–level CATEs, we estimated ATEs at the population level using multiple backdoor–adjustment methods, including regression, stratification, and propensity score weighting (Rosenbaum and Rubin, 1983; Austin, 2011; Chan and Zhang, 2016). We further validated our results through covariate balance checks, model calibration, refutation tests and matching analysis in test data to assess robustness against model misspecification and hidden bias. This comprehensive strategy aligns with best practices in causal inference and supports the development of robust and individualized intervention models (Chernozhukov et al., 2018; Athey and Wager, 2019; Hernán and Robins, 2020). To ensure interpretability, we employed SHAP to analyze model behavior at both the group and individual levels. SHAP summary plots confirmed that clinically relevant features consistently influenced recommendations across the test population. Individual–level force plots provided further transparency, showing how specific features contributed to a patient's recommended setting.

Although the improvement in CMS adherence associated with personalized comfort settings during PAP therapy was modest (4%), the impact could be clinically meaningful at scale. Given the very large sample size, statistical significance is readily attained; furthermore, a formal minimal clinically important difference for comfort–setting–attributable usage gains has not been established. Therefore, the effect size should be interpreted in the context of its timing, scalability, and mode of delivery. Improving adherence to PAP therapy is particularly important during the early treatment period when adherence patterns are formed, and these often predict long–term device usage. Our analysis focused on this timeframe, targeting a window where effective interventions can prevent early dropout and establish consistent PAP usage. Although the observed increase in nightly usage duration was modest, ranging from approximately 12 to 20 min per night, this finding should be interpreted in the context of sustained treatment engagement. At the population level, even small per–patient increases in nightly use may translate into substantial cumulative therapy exposure. In addition, the observed increase in days of use, corresponding to up to 21 additional days of therapy use per year, may be particularly meaningful as it reflects more consistent engagement with treatment rather than only marginal increases in nightly duration.

Importantly, these improvements were achieved through adjustments to comfort settings that are already available within PAP devices, without requiring changes to therapeutic pressure delivery. This suggests that optimisation of existing comfort–related parameters may provide a practical, low–risk opportunity to improve PAP use. These findings are also consistent with dose–response literature indicating that incremental increases in nightly PAP usage are associated with improved health outcomes (Malhotra et al., 2023).

Our findings help reconcile the mixed results reported in previous studies evaluating the impact of PAP comfort settings (e.g., Ramp, EPR, and Soft Response) on patient outcomes. Several key factors likely contribute to this inconsistency. Firstly, many previous studies relied on a uniform intervention approach, applying the same setting configuration to all patients regardless of individual characteristics. This one–size–fits–all strategy fails to account for the heterogeneity in patient responses (i.e. some individuals may benefit significantly from specific comfort settings, while others may experience no benefit or even decreased comfort under the same configuration). As a result, group–level effects often appeared small or statistically insignificant when averaged across a diverse cohort. Secondly, due to small sample sizes, many studies have evaluated each comfort feature in isolation, without accounting for the potential interactions and complimentary nature of multiple settings (Wimms et al., 2013). By utilizing a large and richly–featured dataset of nearly 395,000 patients, the current analysis was able to capture and model these complex interactions, allowing us to assess not only the independent effects of comfort settings but also how they work together to influence adherence. Thirdly, the primary analysis initially evaluated a single PAP device platform and operational mode, which reduced variability arising from hardware differences and proprietary algorithm behavior and enabled a controlled assessment of comfort settings and their impact. However, this limitation is partly addressed by the independent validation analyses, which extended the primary 90–day Air Sense 10 findings to longer–term outcomes and a newer–generation device platform. Specifically, recommendation–matched settings were associated with sustained usage benefits at 1 year and showed consistent effects on the Air Sense 11. These findings support the generalizability of the observed associations to current Resmed hardware.

Overall, by addressing treatment–effect heterogeneity, modeling the interaction between comfort settings, and analyzing data from a uniform hardware and operating mode, our personalized model was able to uncover clinically meaningful improvements that uniform interventions likely obscured.

Some ML models appear as highly complex ‘black box' systems, meaning that they are not user–friendly for medical personnel who may not be well–versed in algorithmic fields (Yang et al., 2024). A key strength of our analysis is the clinical transparency of the model's recommendations. The most influential factors guiding personalized comfort configurations, particularly minimum pressure, ramp start pressure and patient age, are well aligned with how clinicians typically approach PAP therapy. For example, patients started on PAP at higher minimum pressures were frequently recommended settings that improved exhalation comfort, such as enabling EPR. Likewise, a lower ramp start pressure was more commonly recommended for patients struggling with initial pressure tolerance, supporting easier sleep onset. Age was also identified as a consistent driver of settings choice, with older individuals more often receiving comfort–enhancing settings, potentially reflecting differences in pressure sensitivity or sleep physiology.

Overall, our data suggest that the model's logic is clinically aligned and explainable, reflecting patterns that are familiar to experienced sleep clinicians. This interpretability is essential for clinical adoption because it builds trust in model–guided recommendations and supports their use in real–world PAP therapy workflows. The model could be implemented as a clinical decision support tool during device setup, assisting clinicians or respiratory therapists in selecting comfort settings tailored for the characteristics of each individual. Alternatively, the algorithm could be embedded within PAP device firmware or integrated into companion software platforms, enabling automated, personalized configuration during initial therapy initiation or remotely during early follow–up. For real–world deployment, ongoing monitoring would be needed to detect input drift, changes in recommendation patterns, and calibration or treatment–effect instability over time, with human oversight to ensure continued safety and clinical appropriateness.

A key advantage of the model used in this study is its scalability. It leverages features already available on most PAP devices and does not require additional hardware, sensors, or patient–facing tools. This should make it easy to implement, and applicable in both high– and low–touch care environments. Broad adoption has the potential to streamline setup processes, reduce early therapy dropout, enhance patient comfort and satisfaction, and ultimately ease the workload for clinical teams managing adherence issues. By enabling data–driven personalization at scale, this model could support a more proactive and patient–centered approach to OSA therapy.

The physiological effects of comfort features should also be considered when interpreting these findings. EPR lowers delivered pressure during exhalation and may improve comfort, but in susceptible patients it could reduce effective expiratory pressure relative to the pressure needed to maintain airway patency; however, the device–enforced minimum pressure limits the extent of pressure reduction. Soft Response may improve comfort by slowing pressure increases after respiratory events, but this could theoretically delay event resolution in some patients. Because Soft Response was present in only two of the nine modeled comfort–setting combinations and had low prevalence in the cohort, recommendations involving this feature should be interpreted cautiously, particularly in patients with poorly controlled, severe, or position–dependent OSA. Auto Ramp estimates sleep onset using device–derived respiratory signals rather than polysomnographic sleep staging and may delay delivery of full therapeutic pressure at sleep onset; in some patients, this could transiently increase early–night residual events. These considerations highlight that personalized comfort–setting recommendations should complement, rather than replace, clinical assessment of residual events, leak, symptoms, and patient tolerance.

Several limitations should be considered when interpreting the findings of this analysis. Firstly, although the design incorporated robust causal inference methods at both the group and patient level to estimate the treatment effect, some potentially relevant factors were not captured, including socioeconomic status, insurance type, aspects of the patient care pathway (e.g., level of clinical support or coaching intensity, provider expertise), education received about PAP therapy, behavioral or educational interventions, comorbidities including insomnia and psychiatric disease, baseline symptom burden, prior PAP exposure and real–world mask fit or seal performance. AHI was a self–reported metric and therefore is not as accurate as objective diagnostic data, and may have been mistakenly entered as “on treatment AHI” by some users. Although mask type was included as a covariate, we did not account for the exact mask model, which may also influence comfort and adherence. The cohort also did not distinguish central from obstructive apnea, although the model was developed for patients with OSA using the Auto Set algorithm. Overall, while the modeling approach aimed to approximate a randomized framework, the possibility of residual confounding cannot be entirely excluded, and the results should be interpreted as associative and hypothesis–generating. Consistent with all observational designs, propensity score matching and double machine learning mitigate, but cannot completely eliminate, bias arising from unmeasured confounders. Nevertheless, several features of the present analysis constrain the plausibility of substantial residual confounding. The double machine learning estimator is doubly robust to misspecification of either the treatment or the outcome model, and refutation testing was consistent with a genuine effect, as adding a randomly generated common cause left the estimate unchanged (0.0290 vs. 0.0290; *p* = 1.0) whereas a placebo treatment with permuted assignment abolished it. The accompanying E–values indicate that an unmeasured confounder would need to be more strongly associated with both treatment assignment and adherence than the measured covariates already balanced by matching, and the direction and significance of the effect were reproduced across two independent control groups, two device generations, and two follow–up horizons. The estimates are therefore best regarded as robust, hypothesis–generating evidence, while recognizing that only a randomized trial can exclude residual confounding entirely.

Secondly, the cohort selection criteria may have introduced selection bias and survivorship bias. The analysis was restricted to new PAP users assigned to one of nine common comfort–setting combinations who did not modify therapy or comfort settings before achieving target adherence levels within the first 90 days. This restriction was applied to reduce uncontrolled reverse–causation bias during model development; however, it may limit generalizability to patients who adjust settings early or use rarer comfort–setting configurations. Selected and not–selected patients were broadly similar across measured baseline characteristics, with small differences in covariate proportions and mean pressure settings, suggesting that the selection criteria did not introduce meaningful imbalance on observed characteristics. Nevertheless, unmeasured differences may remain, and the underrepresentation of less common configurations, particularly Soft Response settings, introduces uncertainty for recommendations involving these features. Confounding by indication also cannot be excluded, as clinicians may have preferentially assigned certain settings to patients perceived to be at higher risk or to have different clinical needs. In addition, collider bias may be possible if conditioning occurred on variables influenced by both treatment assignment and subsequent outcomes. The retrospective observational design also precludes within–individual comparisons, because no patient was observed under both their actual settings and the model–recommended counterfactual. Therefore, subgroup–specific individual–level causal effects cannot be directly estimated, and prospective crossover or randomized studies would be required to confirm causal effects. These selection and survivorship mechanisms may act on unmeasured as well as measured characteristics and cannot be fully removed by statistical adjustment. Several observations nonetheless suggest that their impact is limited: selected and not–selected patients were broadly similar across measured baseline characteristics, with mean pressure settings differing by less than 0.2 cmH_2_O (Supplementary Table 4); a sensitivity analysis restricted to the four most prevalent comfort–setting combinations remained directionally consistent; and the findings were reproduced in independent, contemporary validation cohorts of more than 1.2 million patients spanning two device generations and one–year follow–up, supporting generalizability beyond the development cohort.

Thirdly, while the primary analysis was developed on a single PAP device platform and operational mode, validation in later Air Sense 10 and Air Sense 11 cohorts supports generalizability to current Resmed hardware. Generalization to non–Resmed devices or different comfort algorithms remains untested. However, the methodological framework, including causal forest estimation, propensity score matching, and SHAP–based explainability, is based on well–established, reproducible methods and can be independently implemented using widely available open–source tools. Bit–for–bit replication by external groups is not possible because the underlying patient–level data are proprietary and the deployed model is a locked, commercially cleared algorithm. The analytical framework is, however, fully specified and reproducible in principle, relying entirely on established open–source libraries (scikit–learn, econml, DoWhy, statsmodels), and the complete technical specification, architecture, training methodology, hyperparameters, and evaluation framework, has undergone independent U.S. FDA review as part of 510(k) clearance, providing additional methodological scrutiny.

Lastly, adherence was assessed using device–reported CMS adherence metrics, which do not fully capture patient–centered outcomes such as improvements in sleep quality, daytime functioning, or overall satisfaction with therapy. The adherence definition also uses US–specific CMS criteria, which may not directly transfer to other health systems or reimbursement contexts. Including these outcomes in future studies will provide a more comprehensive understanding of the impact of personalization. Finally, the low mean minimum pressure observed in this real–world cohort may reflect prescribing patterns or possible under–titration, which a comfort–setting recommendation model alone is not designed to address.

This analysis offers a foundation for several important areas of future research. A key next step is prospective validation of the model in clinical practice. Conducting a randomized controlled trial where patients are assigned to receive either model–guided comfort settings or standard care (e.g., default configurations or clinician–selected settings) would help confirm the causal impact of personalization in a real–world setting. Future analyses should also examine the full continuous adherence distribution (e.g., quantile treatment effects or shifts in the usage–hour distribution) to establish whether gains are concentrated among marginal users near the CMS threshold or distributed across the population.

Beyond validation, future work may consider expanding personalization to additional PAP settings not evaluated in this model. For instance, features such as humidification and tubing temperature, which influence upper airway comfort and tolerance, may benefit from tailored configurations, particularly for people prone to nasal dryness, congestion, or mouth leak. Similarly, incorporating more detailed data on pressure behavior over time could allow fine–tuning of the PAP device pressure response algorithm based on individual respiratory patterns.

Another promising direction involves the development of adaptive, feedback–driven personalization systems. Rather than applying a one–time configuration at setup, intelligent devices or connected platforms could continuously learn from nightly usage patterns and patient–reported experiences. This approach would recognize that patient needs may evolve with experience and acclimatization to therapy.

Finally, the transportability of the personalization model should be explored. Validating its performance across diverse healthcare settings, patient populations, and device ecosystems will be essential to ensuring broad applicability. Integrating patient preferences and shared decision–making into the personalization framework may further enhance engagement and satisfaction.

Continued innovation at the intersection of causal inference and machine learning offers a path toward truly individualized PAP therapy. With further development and clinical integration, these approaches have the potential to meaningfully improve adherence and perhaps also long–term outcomes for people with OSA. These conclusions are drawn from observational data and are best regarded as robust, hypothesis–generating evidence pending prospective randomized confirmation.

## Data Availability

The data analyzed in this study is subject to the following licenses/restrictions the data analyzed in this study are proprietary to Resmed and are not publicly available due to patient privacy, ethical, and commercial confidentiality restrictions. Requests to access these datasets should be directed to Alison Wimms alison wimms@resmed.com.au.
