# Supplementary material for: Personalized comfort settings in positive airway pressure machines using a causal inference approach: a retrospective observational analysis
Source: Front Sleep. 2026 Aug 21;5:1883557. doi: 10.3389/frsle.2026.1883557 (PMC13543805; doi:10.3389/frsle.2026.1883557)
Supplement: Supplementary file 1 [file Supplementary_File_1.docx]

**Personalised PAP comfort settings using causal inference model**

**Supplementary data**

**Figure S1**. Covariate balance measured by standardized mean difference before (unadjusted) and after (adjusted) propensity score matching for secondary analysis

**Figure S1 - Alt text:** Plot of standardized mean differences for covariates before and after propensity score matching, showing reduced imbalance after adjustment, with most adjusted values near zero and within the prespecified balance threshold.

**Figure S2**. SHapley Additive exPlanations (SHAP) summary plot of feature importance across predicted treatments in the test set

**Figure S2** **- Alt text:**  Shapley Additive exPlanations (SHAP) summary plot of mean absolute SHAP values in the test set, ranking features by importance across predicted comfort settings, with minimum pressure and start pressure contributing most to model predictions.

A

B

**Figure S3.** SHapley Additive exPlanations (SHAP) force plots showing feature contributions toward the best treatment prediction for two sample patients: (a) top and (b) bottom

**Figure S3 - Alt text:**  Shapley Additive exPlanations (SHAP) force plots for two patients illustrating how individual features shift the model output from the base value to the predicted treatment score. Red arrows represent features pushing the prediction higher, while blue arrows represent features pushing it lower.

A

B

C

**Figure S4.** The distribution of conditional average treatment effect (CATE) estimates from the causal forest model across (A) age groups , (B) gender categories (and (C) baseline apnea-hypopnea index (AHI) groups

**Figure S4 - Alt text:**  Density plots showing distributions of conditional average treatment effect (CATE) estimates from a causal forest model across age groups, gender categories, and baseline apnea hypopnea index groups, with most estimates centered near zero and varying spread between subgroups.

**Figure S5.** Heatmap of Maximum Standardized Mean Differences (SMD) between any two covariates for each pair of treatment groups
All maximum SMDs are below 0.1 in the test dataset, indicating good covariate balance across treatment groups.

**Figure S5 - Alt text:** Heatmap of pairwise maximum standardized mean differences between treatment groups in the test dataset, with all values below 0.1, indicating good covariate balance across groups.

***Outcome Model***

**Figure S6. Calibration curve of the outcome model**

The curve shows an Expected Calibration Error (ECE) of 0.91% across all predicted probabilities in the test dataset, indicating good model calibration.

**Figure S6 - Alt Text:** Calibration plot of the outcome model in the test dataset showing predicted probabilities closely aligned with observed fractions of positives, with an Expected Calibration Error of 0.91 percent and well distributed prediction scores.

***Final Model***

**Table S1 -** Best Linear Predictor (BLP) estimates for all treatment groups

All BLP estimates are positive, indicating a strong positive relationship between the model’s predicted treatment effects and the actual observed outcomes. The associated p-values confirm that all estimates are significantly different from zero, demonstrating that the final model effectively captures true treatment effect heterogeneity.

| Treatment | | | | | BLP Estimate | Standard Error | P-Value |
| --- | --- | --- | --- | --- | --- | --- | --- |
| Soft Response | **RAMP mode** | **RAMP time** | **EPR mode** | **EPR level** |  |  |  |
| Off | Auto | --- | Full-time | 3 | 0.573 | 0.324 | 0.077 |
| Off | Off | N/A | Full-time | 3 | 0.57 | 0.285 | 0.045 |
| Off | Auto | --- | Full-time | 2 | 0.771 | 0.249 | 0.002 |
| Off | Off | N/A | Full-time | 2 | 0.665 | 0.203 | 0.001 |
| Off | Off | N/A | Off | N/A | 0.605 | 0.165 | 0 |
| Off | Off | N/A | Full-time | 1 | 0.535 | 0.133 | 0 |
| On | Off | N/A | Full-time | 3 | 0.589 | 0.118 | 0 |
| On | Off | N/A | Full-time | 2 | 0.574 | 0.084 | 0 |

**Table S2 -**Treatment-specific average treatment effects for each non-default AirSense 10 comfort-setting combination

| Treatment | ATE | 95% CI |
| --- | --- | --- |
| OFF_AUTO_NA_FULLTIME_THREE | 0.0122 | [0.0121, 0.0124] |
| OFF_OFF_NA_FULLTIME_THREE | 0.0396 | [0.0394, 0.0398] |
| OFF_AUTO_NA_FULLTIME_TWO | 0.0053 | [0.0051, 0.0055] |
| OFF_OFF_NA_FULLTIME_TWO | 0.0429 | [0.0427, 0.0431] |
| OFF_OFF_NA_OFF_NA | 0.0582 | [0.0580, 0.0584] |
| OFF_OFF_NA_FULLTIME_ONE | 0.0485 | [0.0482, 0.0487] |
| ON_OFF_NA_FULLTIME_THREE | 0.0333 | [0.0330, 0.0335] |
| ON_OFF_NA_FULLTIME_TWO | 0.0347 | [0.0344, 0.0349] |

ATE, average treatment effect; CI, confidence interval.

**Table S3 - Sensitivity analysis excluding patients with baseline AHI <5 events/h**

Reports ATE estimates for the original cohort and the AHI ≥5-only cohort across both evaluation frameworks. Excluding patients with self-reported baseline AHI <5 events/h preserved the direction, significance, and approximate magnitude of the estimated treatment effects, indicating that potential misclassification of self-reported baseline AHI did not materially affect the findings. Robustness to broader input uncertainty was additionally assessed in the main-text Monte Carlo input-misclassification analysis.

- Average treatment effect against AirSense 10 default settings

| **Outcome** | **Original (N=5,875)** | **AHI≥5 only (N=3,851)** |
| --- | --- | --- |
| Compliance | +0.045 (p<0.001) | +0.042 (p=0.0004) |
| Duration (min) | +12.2 (p<0.001) | +11.3 (p=0.0002) |
| Usage days | +2.8 (p<0.001) | +2.6 (p=0.0004) |

- Average treatment effect against clinician- or patient-adjusted settings

| **Outcome** | **Original (N=5,875)** | **AHI≥5 only (N=3,851)** |
| --- | --- | --- |
| Compliance | +0.037 (p<0.001) | +0.031 (p<0.001) |
| Duration (min) | +7.6 (p<0.001) | +6.2 (p=0.002) |
| Usage days | +2.0 (p<0.001) | +1.5 (p=0.003) |

**Table S4** - Characteristics of selected and not-selected patients

Compares baseline demographic, clinical, device, and mask-related characteristics between patients included in the nine predefined comfort-setting combinations and those excluded because they used other configurations.

|  |  | Missing | Overall | Selected | Not selected |
| --- | --- | --- | --- | --- | --- |
| n |  |  | 564,000 | 394,919 | 169,081 |
| AGE_GROUP, n (%) | (-Inf, 45) | 0 | 175,551 (31.1) | 122,745 (31.1) | 52,806 (31.2) |
|  | (60, Inf) |  | 148,812 (26.4) | 104,935 (26.6) | 43,877 (26.0) |
|  | [45, 60] |  | 239,637 (42.5) | 167,239 (42.3) | 72,398 (42.8) |
| GENDER, n (%) | Female | 0 | 196,825 (34.9) | 132,355 (33.5) | 64,470 (38.1) |
|  | Male |  | 327,285 (58.0) | 234,126 (59.3) | 93,159 (55.1) |
|  | Prefer not to say |  | 39,890 (7.1) | 28,438 (7.2) | 11,452 (6.8) |
| BASELINE_AHI_GROUP, n (%) | Mild | 0 | 148,962 (26.4) | 105,156 (26.6) | 43,806 (25.9) |
|  | Minimum |  | 107,166 (19.0) | 73,887 (18.7) | 33,279 (19.7) |
|  | Moderate |  | 110,112 (19.5) | 77,532 (19.6) | 32,580 (19.3) |
|  | Severe |  | 125,317 (22.2) | 87,533 (22.2) | 37,784 (22.3) |
|  | Unknown |  | 72,443 (12.8) | 50,811 (12.9) | 21,632 (12.8) |
| MASK_TYPE, n (%) | Full face | 0 | 231,163 (41.0) | 159,005 (40.3) | 72,158 (42.7) |
|  | Nasal |  | 149,638 (26.5) | 106,896 (27.1) | 42,742 (25.3) |
|  | Pillows |  | 183,199 (32.5) | 129,018 (32.7) | 54,181 (32.0) |
| SLEEP_TEST_TYPE, n (%) | Home | 0 | 375,420 (66.6) | 266,495 (67.5) | 108,925 (64.4) |
|  | Lab |  | 188,580 (33.4) | 128,424 (32.5) | 60,156 (35.6) |
| SLEEPINESS_PRE_PAP, n (%) | Extremely | 0 | 20,259 (3.6) | 13,823 (3.5) | 6,436 (3.8) |
|  | Moderately |  | 95,756 (17.0) | 66,004 (16.7) | 29,752 (17.6) |
|  | Not at all |  | 15,597 (2.8) | 10,627 (2.7) | 4,970 (2.9) |
|  | Slightly |  | 46,907 (8.3) | 32,161 (8.1) | 14,746 (8.7) |
|  | Unknown |  | 334,120 (59.2) | 237,030 (60.0) | 97,090 (57.4) |
|  | Very |  | 51,361 (9.1) | 35,274 (8.9) | 16,087 (9.5) |
| MINIMUM_PRESSURE, mean (SD) |  | 0 | 5.4 (1.6) | 5.4 (1.5) | 5.6 (1.8) |
| START_PRESSURE, mean (SD) |  | 0 | 4.2 (0.6) | 4.2 (0.6) | 4.3 (0.6) |
| COMPLIANCE_FLAG, n (%) | 0 | 0 | 116,460 (20.6) | 79,689 (20.2) | 36,771 (21.7) |
|  | 1 |  | 447,540 (79.4) | 315,230 (79.8) | 132,310 (78.3) |

Measured baseline characteristics were similar between selected and not-selected patients; differences in proportions were small and mean pressure settings differed by <0.2 cmH₂O. While similarity on measured characteristics does not fully exclude unmeasured differences, the small observed imbalances, combined with consistent effects in the independent validation cohorts, argue against substantial selection or survivorship bias.

**Table S5** - Sensitivity analysis restricted to the top four comfort-setting combinations

Reports ATE estimates for CMS adherence, nightly usage duration, and usage days when the analysis is restricted to the four most prevalent setting combinations.

- Average treatment effect against AirSense 10 default settings

| **Outcome** | **Original ATE (p)** | **Top-4 ATE (p)** |
| --- | --- | --- |
| Compliance | +0.045 (p<0.001) | +0.033 (p=0.28) |
| Duration (min) | +12.2 (p<0.001) | −1.9 (p=0.81) |
| Usage days | +2.8 (p<0.001) | +3.3 (p=0.07) |

(b) Average treatment effect against clinician- or patient-adjusted settings

| **Outcome** | **Original ATE (p)** | **Top-4 ATE (p)** |
| --- | --- | --- |
| Compliance | +0.037 (p<0.001) | +0.018 (p=0.51) |
| Duration (min) | +7.6 (p<0.001) | −6.0 (p=0.36) |
| Usage days | +2.0 (p<0.001) | +2.2 (p=0.15) |

**Table S6** - Dominant recommended comfort configuration by key patient-feature combinations

| Recommended setting | Typical profile | Clinical logic |
| --- | --- | --- |
| No EPR, no Soft Response | Mid-age, lowest pressure (4.1 cmH₂O), mild AHI, home-tested | Low-pressure patients tolerate therapy without additional pressure-relief assistance |
| EPR level 1, no Soft Response | Mid-age, low pressure (4.3 cmH₂O), unknown AHI, home-tested | Minimal EPR when severity is uncertain |
| EPR level 2 + Soft Response | Younger (<45 years), mid pressure (4.4 cmH₂O), unknown AHI, home-tested | Younger patients with unknown severity receive Soft Response plus moderate EPR |
| EPR level 3 + Soft Response | Mid-age, highest pressure (5.2 cmH₂O), mild AHI, home-tested | Highest-pressure patients receive maximum EPR to aid tolerability |
| EPR level 2, no Soft Response | Mid-age, high pressure (4.9 cmH₂O), mild AHI, laboratory-tested | Laboratory-diagnosed, high-pressure patients receive EPR without Soft Response |

AHI, apnea-hypopnea index; EPR, expiratory pressure relief. Profiles summarize dominant model recommendations and are descriptive rather than deterministic rules.
